# Thraustochytrids from the Red Sea mangroves in Egypt and their abilities to produce Omega-3 fatty acids

**DOI:** 10.1186/s12934-026-03120-9

**Published:** 2026-10-11

**Authors:** Mahmoud D. Abdelkahar, Mahmoud S. Bakhit, Ashraf F. El-Baz, Mohamed A. Abdel-Wahab

**Affiliations:** 1 Department of Botany and Microbiology, Faculty of Science, Sohag University, Sohag, 82524 Egypt; 2 Department of Industrial Biotechnology, Faculty of Biotechnology, University of Sadat City, Sadat City, Egypt

**Keywords:** Egyptian mangroves, GC-FID, Lipids, Marine microbes, Molecular phylogenetics, Oleaginous microbes, Protists, PUFAs

## Abstract

Forty-one isolates of the genus *Aurantiochytrium* were cultured from 32 samples collected from the Red Sea mangroves, Egypt. Cultured thraustochytrids isolated from: decaying leaves of *Avicennia marina* (34 isolates), decaying pneumatophores of *A*. *marina* (3 isolates), and decaying leaves of *Zostera marina* (4 isolates). Of the 41 strains, three strains (SUMCC 21014, SUMCC 21015, and SUMCC 21016) were selected based on both their high biomass production and high lipid content. Phylogenetic analyses based on 18 S rDNA placed the three strains within the *Aurantiochytrium* clade with high statistical support. The three strains were cultured on GYP broth (glucose, yeast extract, peptone) and exhibited different dry cell weights and lipid contents. Lipid profiles were determined using GC-FID analysis. Among the three studied strains, SUMCC 21016 produced the highest biomass of 2.04 g/L, the highest lipid content (0.991 g/L), and a good docosahexaenoic acid percentage (0.217 g/L). Among the three studied strains, SUMCC 21014 produced the highest percentages of DHA (30.3%) and EPA (5.45%) of the total fatty acids but its dry weight is lower than SUMCC 21016.

## Introduction

Thraustochytrids are unicellular, saprobic microorganisms that found exclusively in marine habitats [1]. They are abundant in regions with high amounts of detritus and decaying plant material, especially in mangroves. In recent years, they received more interest as producers of many value-added compounds, such as polyunsaturated fatty acids (PUFAs), carotenoids, squalene, and industrial enzymes [2–6].

Thraustochytrids were first discovered in 1934 by F. K. Sparrow [7], then they have been reported from different locations around the world; Egypt and Malaysia [8], India [9], Micronesia [10], Japan [11], Hong Kong [12, 13], Australia [14], Malaysia [15], England [16], USA [17], and Saudi Arabia [18].

Mangroves are the best source for thraustochytrids because they are rich in fallen, decomposed leaves, and represent one of the most carbon-rich ecosystems in the world [19]. Thraustochytrids prefer high carbon concentration for growth and a high carbon-to-nitrogen (C: N) ratio to accumulate lipids [20]. They represent the major part of the microbiota in the mangrove ecosystems [21, 22]. Thraustochytrids are not only the first colonizers of the fallen mangrove leaves, but they may serve as a food source for other organisms in their habitats due to their high content of essential lipids [21–24].

In 1986, Ulken [8] examined the presence of thraustochytrids in the sediment of two mangrove swamps: one located on the Red Sea coast of Egypt, between El-Quseir and Safaga, and the other near Port Klang in Malaysia. In the Egyptian mangrove, she recorded 22,800 to 65,700 infective units of the genera *Thraustochytrium* and *Schizochytrium* per liter of sediment. However, since her study no other reports of thraustochytrids from Egypt. The present study aims to isolate thraustochytrids from the Egyptian mangroves, describe their morphological and molecular characteristics, and investigate their abilities to produce omega-3 fatty acids.

## Materials and methods

### Sample collection and isolation of thraustochytrids

On 3 November 2021, a total of 32 samples were collected from four mangroves sites along the Red Sea coast in Egypt namely: 17 km south of Safaga city (26°36ʹ58ʺ N, 34°00ʹ36ʺ E), 45 km south of Safaga city (26°23ʹ55ʺ N, 34°07ʹ15ʺ E), 35 km south of El Quseir city (25°52ʹ02ʺ N, 34°24ʹ53ʺ E), and 40 km south of El Quseir city (25°50ʹ05ʺ N, 34°26ʹ27ʺ E). Samples included: 20 decaying leaves of *Avicennia marina* (Forssk.) Vierh. at different stages of deterioration, five decaying pneumatophores of *A*. *marina*, and seven decaying leaves of the seagrass *Zostera marina* L. Plant materials of *Avicennia marina* and *Zostera marina* were identified and deposited at the Sohag University Botanical Herbarium (Institutional acronym: SHG). The voucher specimens were assigned the numbers SHG-2021-011 (*Avicennia marina*) and SHG-2021-012 (*Zostera marina*) and are publicly accessible for verification. The processing of samples at the laboratory and the isolation of thraustochytrids were previously described [18].

Collected samples were either: (1) Placed in sterile 50 mL falcon tubes containing 25 mL of 50% seawater dusted with heat-sterilized pine pollen grain (in a hot air oven at 110 °C for 3 days), or (2) placed in clean plastic bags and processed at the laboratory within 24 h. Samples from the plastic bags were washed using sterile seawater, cut into 1 cm long pieces, and then washed in sterile seawater containing 0.5 g/L chloramphenicol to inhibit bacterial growth. These pieces were subsequently transferred into petri dishes containing GYPA medium (5 g glucose, 2 g yeast extract, 2 g peptone, 20 g artificial sea salt, 0.5 g chloramphenicol, and 20 g agar in 1 L). Samples from falcon tubes were incubated at 25 °C for 2 days. After incubation, the tubes were shaken, and 100 µL of the sample was transferred and spread onto the surface of GYPA plates. The plates were then incubated at 25 °C and examined daily for the presence of growing colonies.

Thraustochytrid colonies were transferred to new GYPA petri dishes until a pure culture was obtained. Thraustochytrid isolates were preserved on GYPA slants (30 g glucose, 5 g peptone, 5 g yeast extract, 20 g artificial sea salt, and 20 g agar in 1 L) and subcultured every two months. Additionally, autoclaved cryotubes containing 1.8 mL of a 50% seawater: glycerol (90:10, v: v) mixture were inoculated with the pure isolates and incubated at 4 °C for 30 min for soaking, then preserved at -80 °C [25]. Thraustochytrid isolates were deposited at Sohag University microbial culture collection (SUMCC), Egypt.

### DNA sequencing and phylogenetic analysis

The three thraustochytrid strains were grown in GYP broth, and the resulting cells were centrifuged at 4000 rpm for 10 min. The supernatants were discarded, and the cells were washed twice in sterile distilled water and centrifuged again. DNA was extracted using the Microbial DNA extraction kit (MOBIO; Mo Bio Laboratories) according to the manufacturer’s instructions. The primer pairs 18S001 and 18S13 were used for PCR amplification and sequencing of the partial 18 S rDNA gene [26]. PCR amplification and sequencing were performed using the methods described by Abdel-Wahab et al. [27], and conducted by Solgent Co., Ltd. (South Korea). Sequencher 4.2.2 (Gene Codes Corporation) was used to assemble the sequence, and ClustalX [28] was used to align it with pertinent sequences obtained from GenBank. Phylogenetic analyses were performed based on maximum likelihood (ML), maximum parsimony (MP), and Bayesian inference (BI). The ML analysis was performed using RAxML GUI v. 2.0.13 [29], the MP analysis by PAUP* 4 [30], and the BI analysis was conducted in MrBayes v. 3.1.2 [31], with details as outlined by Bakhit & Abdel-Wahab [32, 33]. The newly generated nucleotide sequences were deposited in the GenBank database.

### Lipid extraction and fatty acids analysis

#### Culture composition and microbial propagation

Each strain was inoculated into 50 mL of GYP broth (15 g/L glucose, 5 g/L yeast extract, 5 g/L peptone, 20 g/L salt) in 250 mL glass conical flasks and incubated in a shaking incubator at 160 rpm at 25 °C for 48 h. Following incubation, fresh flasks containing the same medium were inoculated with 5% of the 48-hour-aged culture. These flasks were then incubated under the same conditions for 5 days. After this incubation period, the cells were harvested by centrifugation at 4000 rpm, washed twice with distilled water, and subsequently dried at 60 °C in a hot air oven for 24 h [34, 35].

### Lipid extraction method

A modified method was applied to extract lipids from thraustochytrids, as described by Byreddy et al. [34]. Because of the unavailability of freeze drier, a modification of the method was the replacement of the extraction from the freeze dried cells by dried cells in oven at 60 °C for 24 h. Hexane was added in a ratio of 3 mL/ 50 mg dried biomass and ground with a glass rod until it became powder, then vortexed for 2 min. Tubes were centrifuged at 4000 rpm for 15 min. After centrifugation, the supernatant was carefully collected in a pre-weighed glass vial and hexane was evaporated at room temperature. The lipid content percentages were determined by weighing the glass vial after solvent evaporation. Lipid content percentage = mass of lipid/mass of cells *100.

### Gas chromatography-flame ionization detector analysis

Fatty acid methyl esters (FAME) were prepared and analyzed at the Regional Center for Food and Feed in Cairo, Egypt, according to Jøstensen & Landfald [36]. Fatty acid composition was determined using PerkinElmer (Waltham, MA) Clarus-580 GLC equipped with a flame ionization detector (FID), and an HP88 capillary column (30 m × 0.25 mm i.d, 0.20 μm film thickness). GLC-461 (NuChek Prep, Inc., Elysian, Minnesota, USA) was used as the reference standard to quantify fatty acids with carbon chain lengths ranging from C12 to C24. The injection volume was one µL (10:1 split ratio), and helium was used as a carrier gas at a constant flow rate of 15 mL/min. The column oven temperature was initially set at 100 °C and then increased at a rate of 10 °C/min to 220 °C, where it was kept for 15 min. The temperatures of the injector and detector were maintained at 240 °C and 280 °C, respectively with an injection volume was one µL (10:1 split ratio). Methyl ester fatty acid peaks were identified by comparison with reference standards, which were injected under the same conditions.

## Results

### Diversity of thraustochytrids

A total of 41 isolates of thraustochytrids were cultured from 32 samples collected from the Red Sea mangroves, Egypt. All isolated thraustochytrids belonged to the genus *Aurantiochytrium*. Cultured thraustochytrids isolated from decaying leaves of *Avicennia marina* (34 isolates), decaying pneumatophores of *A*. *marina* (3 isolates), and decaying leaves of *Zostera marina* (4 isolates). Of the 41 *Aurantiochytium* strains, three (SUMCC 21014, SUMCC 21015, and SUMCC 21016) were selected based on their high biomass productivity and high percentages of lipids for more morphological and physiological investigation.

### Molecular and morphological identification of *Aurantiochytrium* strains

Phylogenetic analysis based on 18 S rDNA sequencing was employed to confirm the identification of the three *Aurantiochytrium* strains. Three molecular algorithms, namely: Maximum Likelihood (ML), Maximum Parsimony (MP), and Bayesian Inference (BI), were used in the molecular identification of the *Aurantiochytrium* isolates. Morphological identification of *Aurantiochytrium* species was done based on colony color (pale-yellow, orange, white creamy) on plates, sporangia (globose, subglobose, pear-shaped), ectoplasmic net (present, absent), zoospores (released by partial dissolution, complete dissolution, through a pore), amoeboid cells (present, absent), and successive binary divisions (present, absent).

### Phylogenetic analyses of *Aurantiochytrium* strains

The dataset comprised 45 thraustochytrid strains, of which 24 belong to *Aurantiochytrium*, 11 are representatives of other genera of thraustochytrids with *Bacillaria paxillifer* (MG799152) and *Ochromonas danica* (JQ281514) were used as the outgroup. The maximum parsimony dataset consists of 602 characters, including 279 constant, 72 variable parsimony-uninformative, and 251 parsimony-informative characters. MP analysis resulted in 2 most parsimonious trees with a tree length of 604 steps, a consistency index (CI) of 0.6192, a retention index (RI) of 0.8157, and a rescaled consistency index (RC) of 0.5051.

**Fig. 1:**
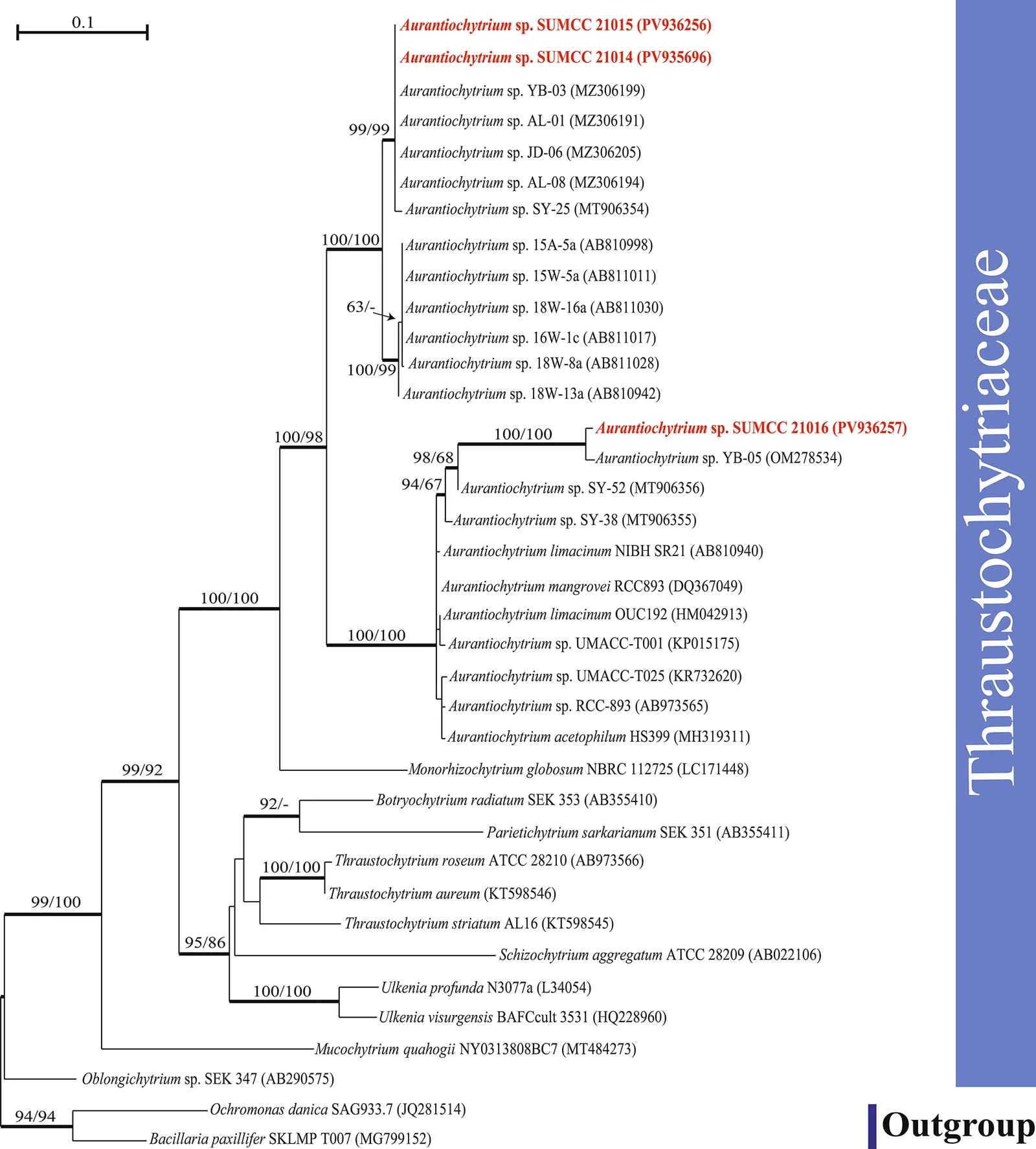
Phylogram generated from ML analysis (RAxML) based on an 18 S rDNA sequence dataset for *Aurantiochytrium* sp. (SUMCC 21014, 21015, 21016) along with other strains and species of *Aurantiochytrium* and other related genera. Bootstrap support on the nodes represents ML, and MP ≥ 60%. Branches with a BI of ≥ 95% are in bold. Newly generated sequences are indicated in red

The ML analysis of the 18 S rDNA dataset yielded the best-scoring tree with a final ML optimization likelihood value of -4517.138716 which is shown in Fig. 1. The matrix contained 327 distinct alignment patterns, with 6.92% of the characters being undetermined or gaps. Estimated base frequencies were found to be A = 0.259198, C = 0.204924, G = 0.274186, T = 0.261692; substitution rates, AC = 0.673691, AG = 1.840526, AT = 1.048394, CG = 1.364357, CT = 2.496324, GT = 1.00; proportion of invariable sites, I = 0.000100 and gamma distribution shape parameter α = 0.504732. The Bayesian analysis resulted in 50,000 trees after five million generations. The topologies obtained from the three analyses were similar. Our phylogenetic analysis placed the new strain of *Aurantiochytrium* sp. (SUMCC 21016) with *Aurantiochytrium* sp. YB-05 (OM278534) in one clade and placed the new strains of *Aurantiochytrium* sp. (SUMCC 21014 and SUMCC 21015) with *Aurantiochytrium* strains YB-03 (MZ306199), AL-01 (MZ306191), JD-06 (MZ306205), AL-08 (MZ306194), and SY-25 (MT906354) in another clade. The 18 S rDNA sequences of *Aurantiochytrium* sp. (SUMCC 21014, 21015, and 21016) were submitted to GenBank under the accession numbers PV935696, PV936256, and PV936257, respectively (Fig. 1).

### Morphological characteristics of *Aurantiochytrium* strains

- *Aurantiochytrium* sp. (SUMCC 21014)

**Fig. 2:**
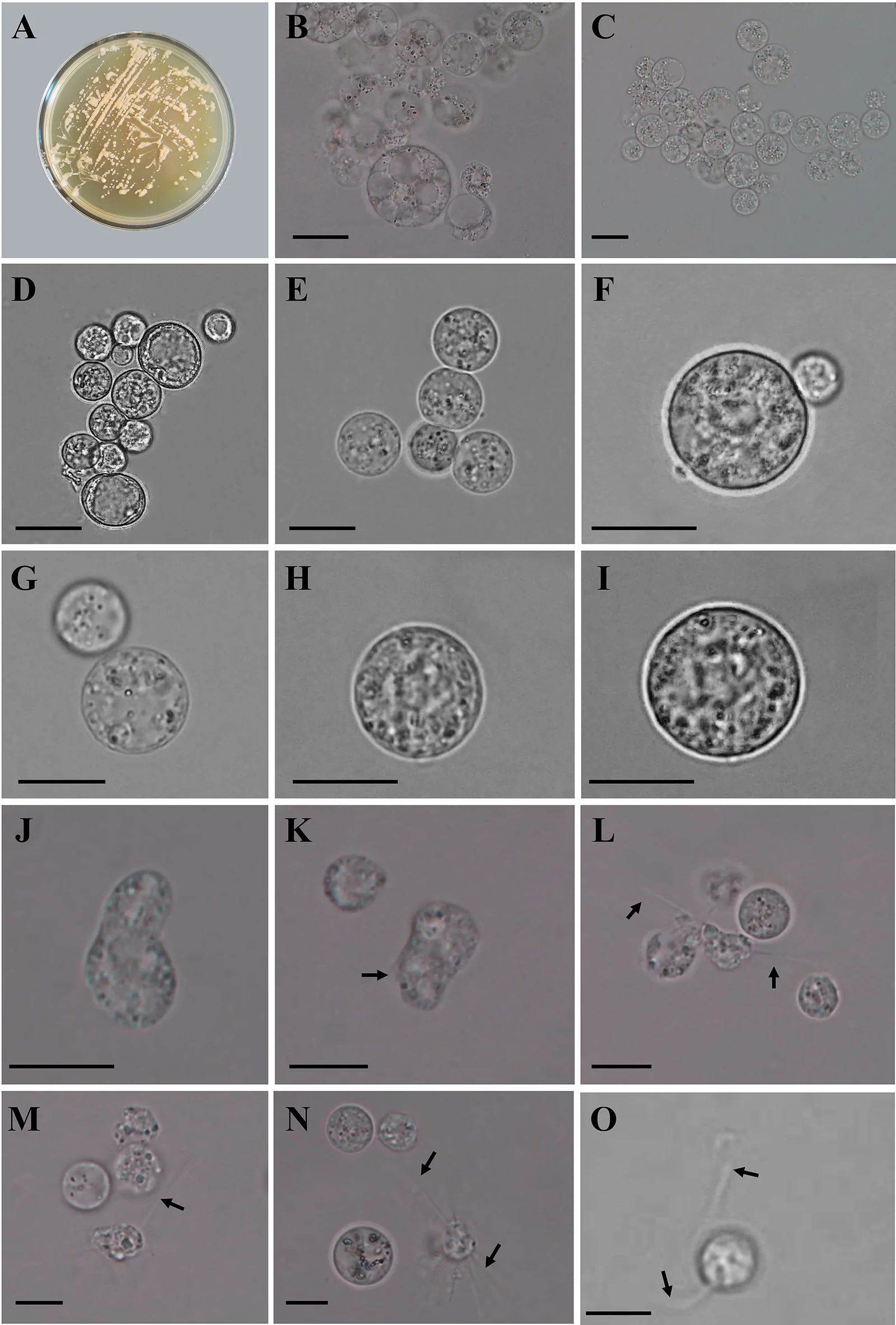
*Aurantiochytrium* sp. (SUMCC 21014). **A** pure culture on GYPA medium. **B**–**I** vegetative sporangia. **J** and **K** amoeboid cells (arrowed in **K**). **L**, **M** sporangia with ectoplasmic appendages (arrowed). **O** zoospore with flagella (arrowed). Scale bars: **B** -* N* = 10 μm, **O** = 5 μm

Colony color is pale-yellow to pale-pink, vegetative cells 7–18 μm, hyaline to pale-pigmented, globose or subglobose, thin-walled, bipartition not observed. Zoospores 4 × 5 μm, biflagellate, pyriform, globose or subglobose. Ectoplasmic appendages were observed. Amoebae 9–15 μm, hyaline to pale-pigmented (Fig. 2).

- *Aurantiochytrium* sp. (SUMCC 21015)

**Fig. 3:**
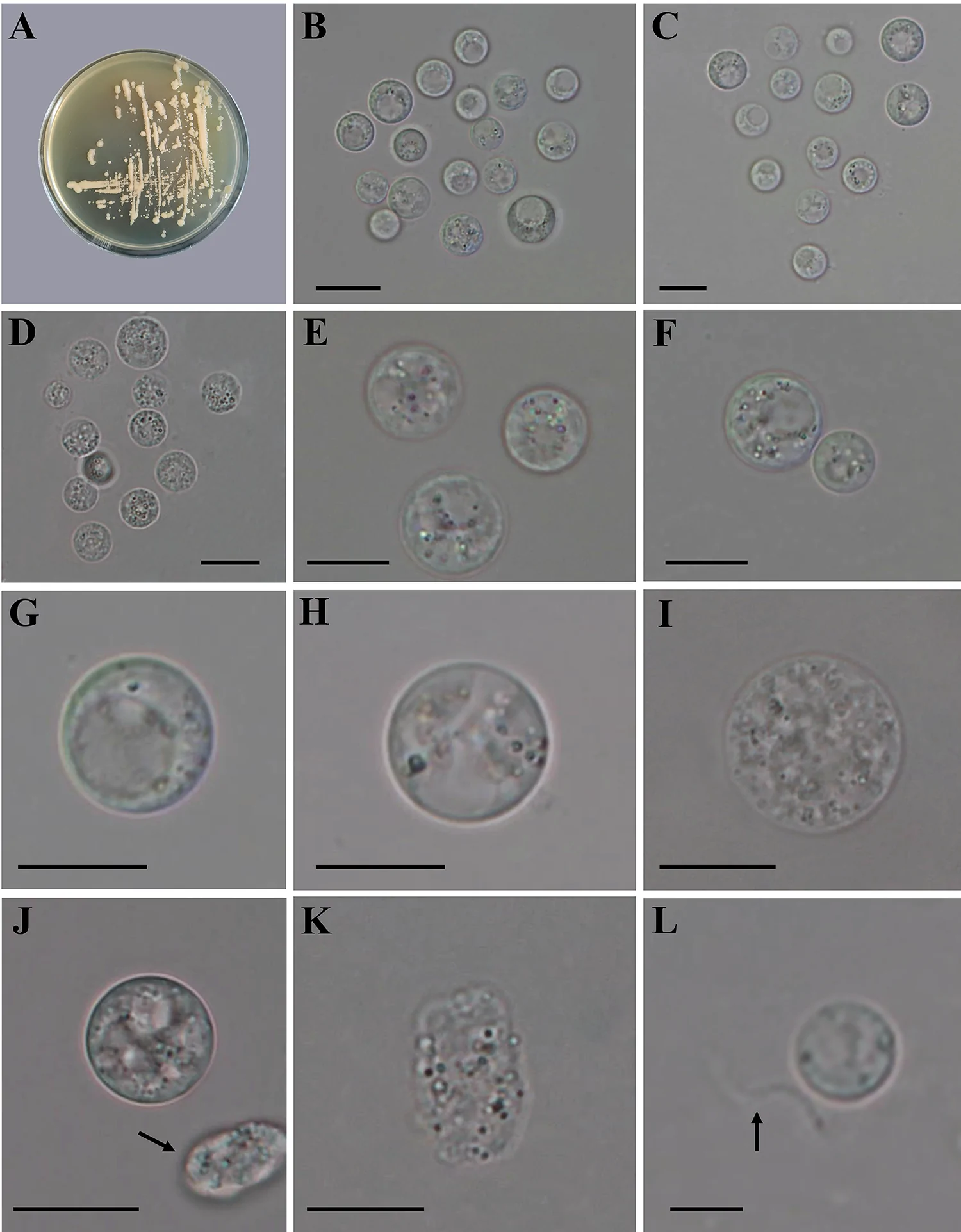
*Aurantiochytrium* sp. (SUMCC 21015). **A** pure culture on GYPA medium. **B**-**I** vegetative sporangia. **J** and **K** amoeboid cells (arrowed in **J**). **L** zoospore with a flagellum (arrowed). Scale bars: **B-K =** 10 μm, **L** = 2 μm

Colony color pale-yellow to pale-orange, vegetative cells 5–14 μm, hyaline to pale-pigmented, globose or subglobose, thin-walled, bipartition not observed. Zoospores 2 × 2.5 μm, biflagellate, irregular in shape or pyriform. Amoebae 10–21 μm, hyaline to pale-pigmented, irregular in shape (Fig. 3).

- *Aurantiochytrium* sp. (SUMCC 21016)

**Fig. 4:**
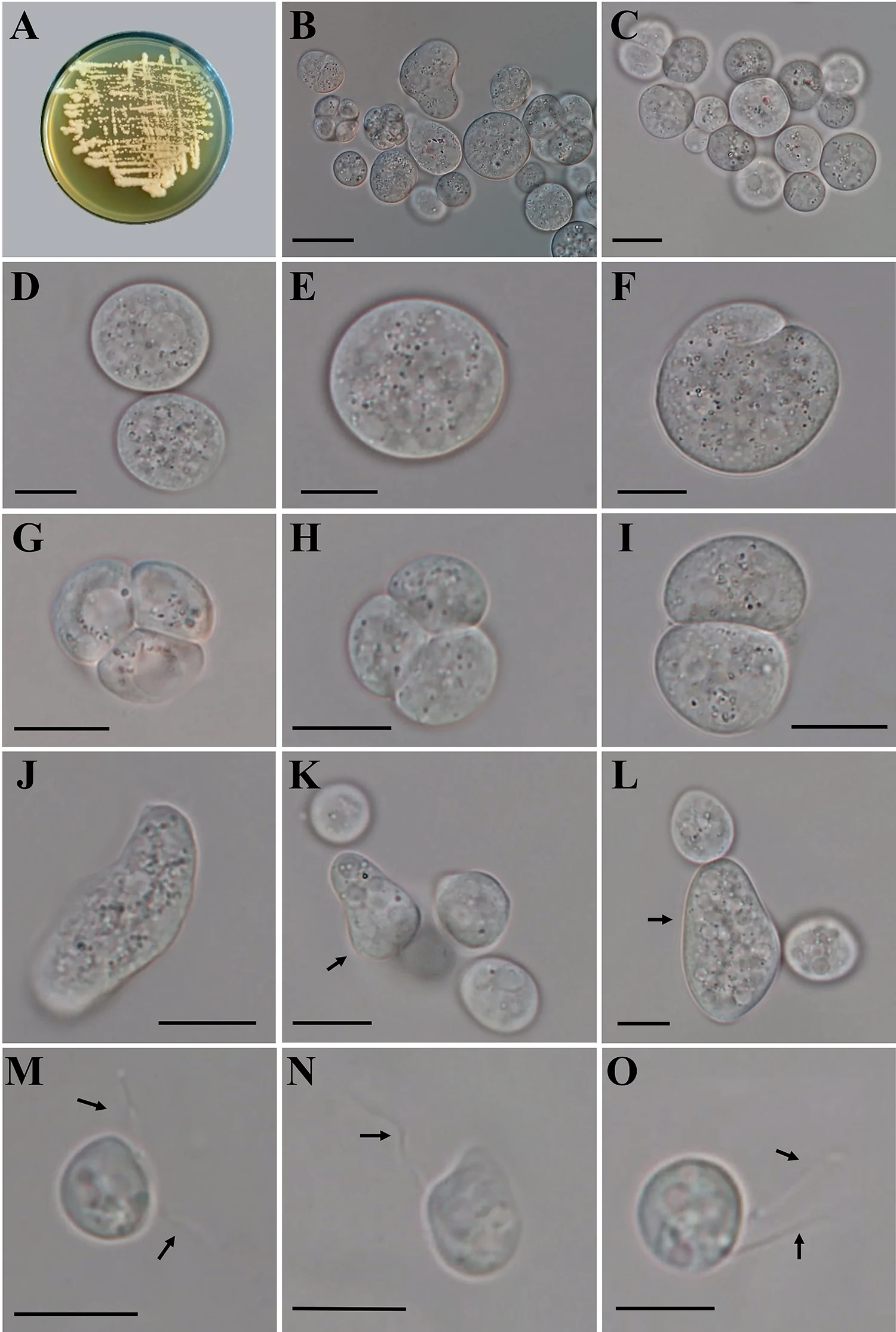
*Aurantiochytrium* sp. (SUMCC 21016). **A** pure culture on GYPA medium. **B**-**F** vegetative sporangia. **G**-**I** bipartitions in cell clusters. **J-L** amoeboid cells (arrowed in **K** and **L**). **M** and **N** active fusiform zoospores with flagella (arrowed). **O** resting zoospore with flagella (arrowed). Scale bars: **B** and **C** = 20 μm, **D-O** = 10 μm

Colony color is white creamy to pale-yellow, vegetative cells 10–25 μm, pale-pigmented, globose or subglobose, thin-walled, and bipartition commonly occurs. Zoospores 5–10 × 12–15 μm, biflagellate, irregular in shape, pyriform or fusiform (while moving) to globose or subglobose (at resting). Amoebae 14–30 μm, pale-pigmented, irregular in shape (Fig. 4).

### Biomasses and lipid profiles of *Aurantiochytrium* strains

**Fig. 5:**
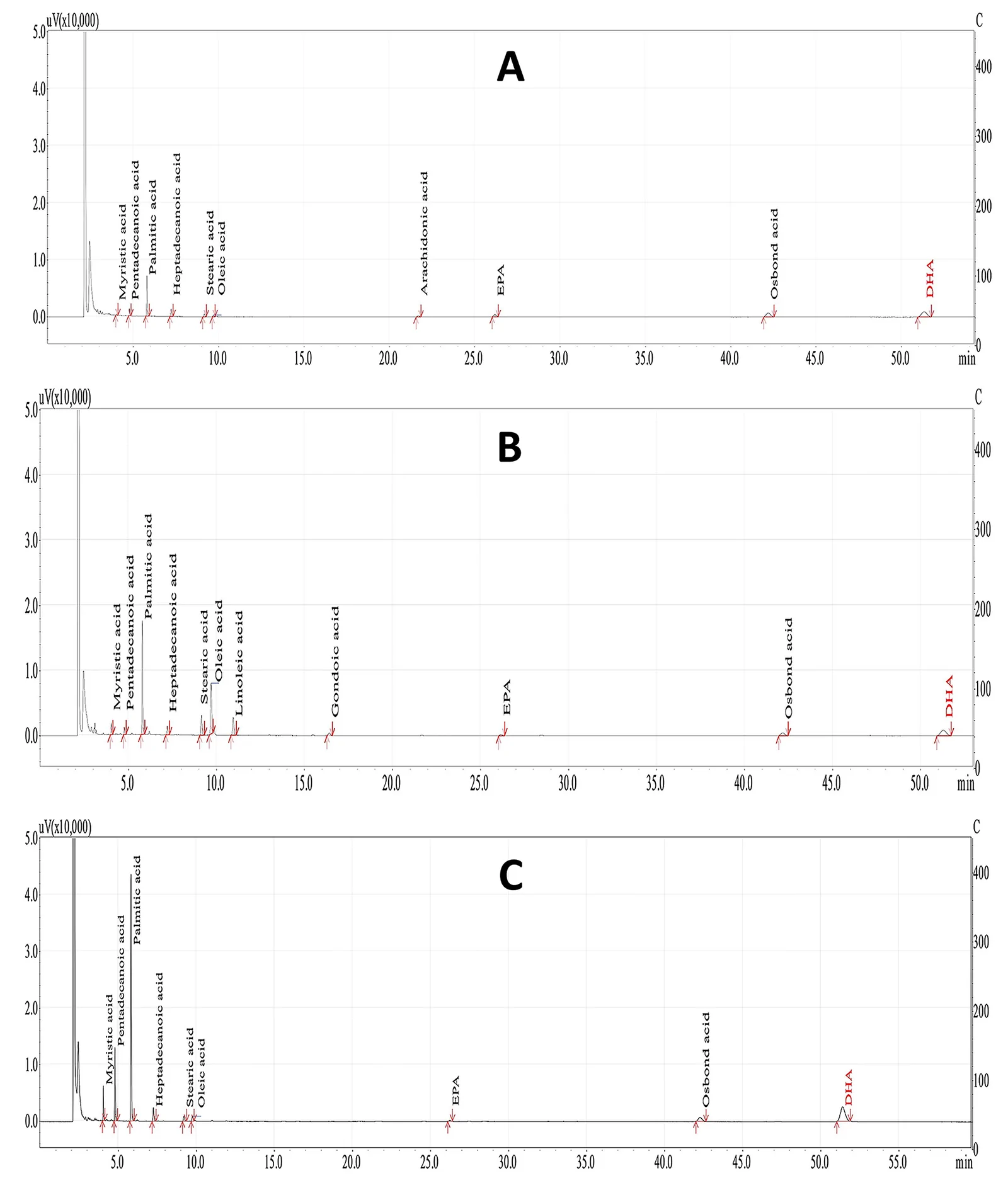
GC-FID chromatographs of *Aurantiochytrium* sp. **A** (SUMCC 21014), **B** (SUMCC 21015), and **C** (SUMCC 21016) cultured on glucose as a carbon source

*Aurantiochytrium* sp. (SUMCC 21016) achieved a higher biomass of 2.04 g/L and a higher lipid content (48.6%) than *Aurantiochytrium* sp. (SUMCC 21014) (0.35 g/L biomass, 27.7% TFA) and *Aurantiochytrium* sp. (SUMCC 21015) (0.204 g/L biomass, 15.4% TFA) (Fig. 5)

**Fig. 6:**
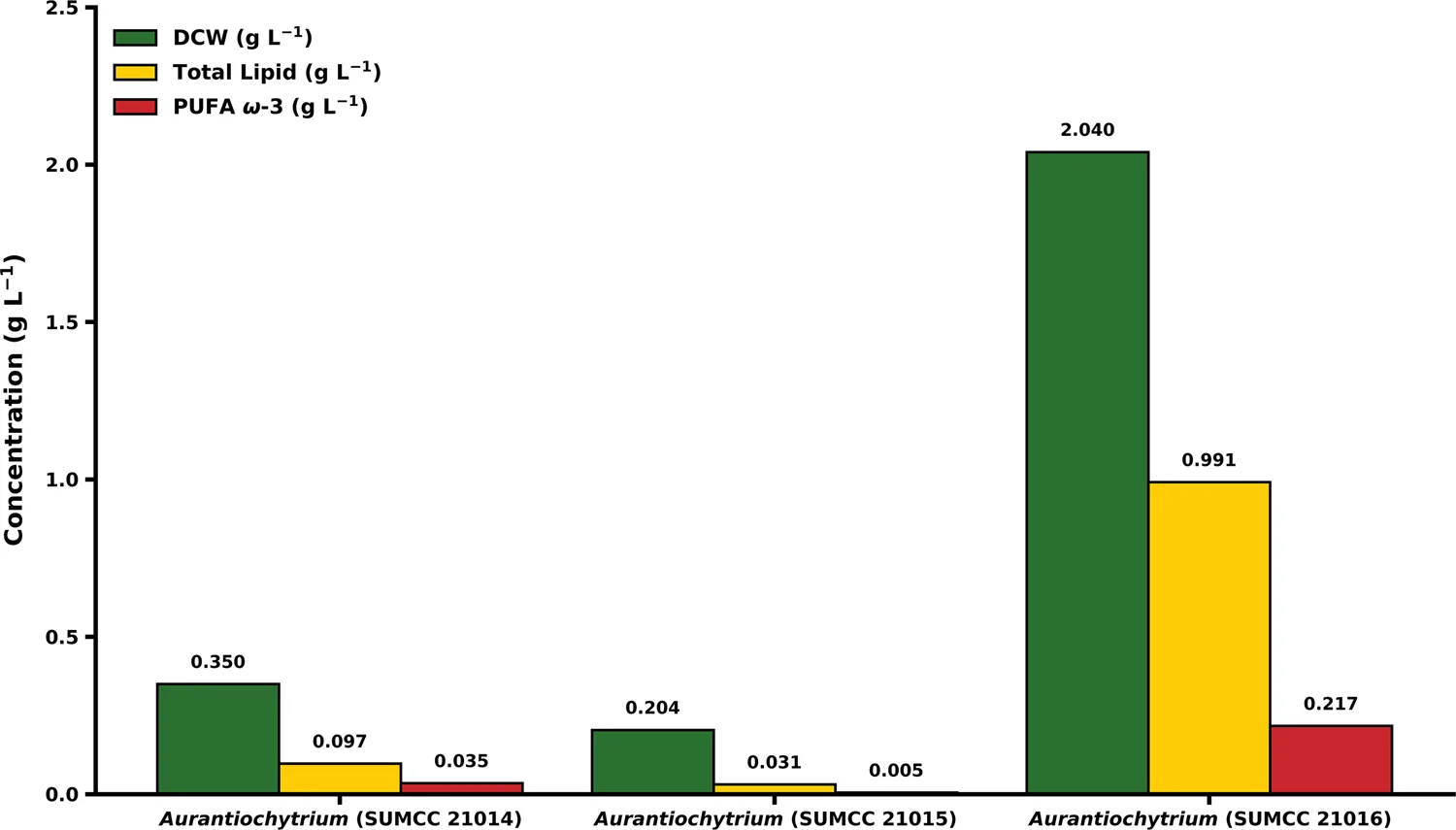
Dry cell weight, lipid content, and omega-3 PUFA in g/L of the three studied *Aurantiochytrium* strains

Glucose represents the standard carbon source for thraustochytrids cultivation. Table 1 and Figure 6 show a comparison between the three strains in terms of dry cell weight (DCW), lipid content, and the fatty acid composition of the lipid contents, while Table (2) presents the various outcomes of utilizing glucose as a carbon source for docosahexaenoic acid (DHA) synthesis by *Aurantiochytrium* species. Our findings were consistent with these results in terms of the total fatty acids (TFA) percentage and the concentration of DHA in the lipid profile. Nevertheless, further optimization is needed to enhance the biomass yield.

The extracted lipid contents were analyzed by GC-FID to determine their fatty acid composition. Lipid profile of *Aurantiochytrium* sp. (SUMCC 21014) contained 30.3% DHA, 28.16% palmitic acid, 17.5% osbond acid, 6.5% heptadecanoic acid, and 5.45% eicosapentaenoic acid (EPA). Strain (SUMCC 21015) have 31.4% palmitic acid, 22.71% oleic acid, 9.15% linoleic acid, and a low DHA percentage of 12.81% in its lipid content. Palmitic acid was also dominant in strain (SUMCC 21016) (46.93%), followed by DHA, pentadecanoic, myristic, and osbond acids (21.21%, 12.95%, 5.51%, and 4.9%, respectively).

**Table 1:** Dry cell weight (g/L), lipid percentage in DCW, fatty acids percentages in lipid content (%) of the three studied strains of *Aurantiochytrium*

DCW (g/L)		Aurantiochytrium SUMCC 21,014	Aurantiochytrium SUMCC 21,015	Aurantiochytrium SUMCC 21,016
		0.35	0.204	2.04
Lipid % DCW		27.7	15.4	48.6
Fatty acids	Percentage of fatty acids in lipid content			
Saturated fatty acids (SFA)				
Myristic acid	C14:0	0.76	2.55	5.51
Pentadecanoic acid	C15:0	4.42	1.76	12.95
Palmitic acid	C16:0	28.16	31.4	46.93
Heptadecanoic acid	C17:0	6.5	3.01	3.9
Stearic acid	C18:0	2.83	8.56	1.98
Monounsaturated fatty acids (MUFA)				
Oleic acid	C18:1 ω-9	1.36	22.71	1.94
Gondoic acid	C20:1 ω-9	-	1.99	-
Polyunsaturated fatty acids (PUFA)				
Linoleic acid	C18:2 ω-6	-	9.15	-
Arachidonic acid	C20:4 ω-6	2.71	-	-
Osbond acid (ω-6 DPA)	C22:5 ω-6	17.5	4.62	4.9
Eicosapentaenoic acid (EPA)	C20:5 ω-3	5.45	1.43	0.69
Docosahexaenoic acid (DHA)	C22:6 ω-3	30.3	12.81	21.21
Total SFA		42.67	47.28	71.27
Total MUFA		1.36	24.70	1.94
Total PUFA ω-6		20.21	13.77	4.90
Total PUFA ω-3		35.75	14.24	21.90

## Discussion

The present study aimed to isolate and identify thraustochytrids from Red Sea mangrove in Egypt, analyze their lipid composition, and evaluate their potential for omega-3 production. A total of 41 isolates were recovered from four mangrove sites along the Red Sea coast in Egypt. They were isolated from decaying leaves and pneumatophores of *Avicennia marina*, and decaying leaves of *Zostera marina*. Thraustochytrids were mainly isolated from estuarine and marine waters, mangrove litter and debris, algae, and sediments [1, 18]. They play a significant ecological role in these habitats through the secretion of extracellular enzymes, such as cellulases and xylanases, which facilitate the degradation of plant-derived substrates including algal tissues and mangrove leaf litter [37]. All the isolates obtained in this study belonged to the genus *Aurantiochytrium*. Yokoyama and Honda [38] established the genus *Aurantiochytrium* to accommodate *Schizochytrium limacinum* and *S. mangrovei* that are distinguished from other *Schizochytrium* species by possessing not only β-carotene but also astaxanthin, phoenicoxanthin, and canthaxanthin, less than 5% of arachidonic acid, compared to *Schizochytrium* that contains about 20% arachidonic acid. Ectoplasmic net is not well-developed in *Aurantiochytrium* species, contrary to *Schizochytrium* species which have well-developed structures. Later on, Ganuza et al. [17] described *Aurantiochytrium acetophilum* as a new species and introduced the first evidence of sexual reproduction in thraustochytrids without a detailed description of the process. The three strains of this study were morphologically and physiologically (based on the lipid profile) consistent with the description of the genus. *Aurantiochytrium* species are generally abundant in mangrove sites, in addition, the three *Aurantiochytrium* species were isolated from mangrove areas [9, 10, 17]. Phylogenetic analysis based on 18 S rDNA placed our strain SUMCC 21,016 with another *Aurantiochytrium* strain (YB-05), while the two other strains (SUMCC 21014, and SUMCC 21015) were grouped with five *Aurantiochytrium* strains (Al-01, Al-08, YB-03, SY-25, and JD-06). All of these strains were isolated from the Red Sea mangroves in Saudi Arabia [18, 25].

In the present study, different fatty acids were reported, however, the important fatty acids were pentadecanoic acid, docosahexaenoic acid, eicosapentaenoic acid, docosapentaenoic acid, linoleic acid, palmitic acid, and oleic acid. Pentadecanoic acid (C15:0) is a monounsaturated omega-7 fatty acid. It occurs in the biomass of the three *Aurantiochytrium* strains. Several studies have highlighted the vital role of this fatty acid in supporting liver and cardiometabolic health [39, 40]. Indeed, pentadecanoic acid was reported to have anti-inflammatory and anti-anemia properties [39, 41, 42]. The fatty acid also induces glucose uptake and increases insulin-sensitivity [43]. Docosahexaenoic acid (DHA), eicosapentaenoic acid (EPA), docosapentaenoic acid (DPA ω-6 and ω-3), and linoleic acid are essential polyunsaturated fatty acids (PUFAs) that recorded in considerable amount in the three studied strains. DHA was a major component of the TFA in the *Aurantiochytrium* strains SUMCC 21014, and 21016, comprising 30.3%, and 21.21%, respectively. *Aurantiochytrium* (SUMCC 21014) contained a higher EPA content (5.45%) compared to the other strains (> 2%), and a higher osbond acid content (17.5%) compared to approximately 4% in strains SUMCC 21015, and 21016.

Docosapentaenoic acid has two isomers: ω-6 DPA with the trivial name osbond acid and ω-3 DPA (clupanodonic acid), and both of them have anti-inflammatory and other beneficial properties [44, 45]. Linoleic acid (C18:2) is a polyunsaturated omega-6 fatty acid that reduces cardiovascular diseases, LDL, and total cholesterol [46], and has antibacterial properties [47, 48]. Linoleic acid represented 9.15% of the TFA of *Aurantiochytrium* sp. (SUMCC 21015) that was cultured on glucose. PUFAs are known for their neuroprotective, cardioprotective, and hepatoprotective properties [49–51]. They are also integrated into red blood cells and plasma phospholipids in humans [52]. These fatty acids also contribute to protection against tumors and autoimmune diseases, such as osteoarthritis [53]. Indeed, PUFAs provide antioxidant, antihypertensive, antidepressant, and antiaging activities [54]. The global docosahexaenoic acid (DHA) market size was projected to be USD 4.55 billion in 2024 and is expected to reach USD 10.06 billion by 2033 with a CAGR of 9.2% during the forecast period from 2025 to 2033 [55].

Palmitic acid (C16:0), also known as hexadecanoic acid, is a saturated fatty acid that was recorded at percentage ranged between 28.16% (SUMCC 21014) and 46.93% (SUMCC 21016) (Table 1). This fatty acid has antioxidant, anticancer, antimicrobial, nematicidal, hemolytic, anti-androgenic, antidiabetic, hypocholesterolemic, and pesticidal properties [56–60]. *Schizochytrium* sp. S056 was used in biodiesel feedstock production due to its high content of palmitic acid [61]. Palmitic acid derived from *Aurantiochytrium* strains is promising for biodiesel production due to its high cetane number, good oxidation stability, and low iodine value [18, 62].

Oleic acid (C18:1) is a monounsaturated omega-9 fatty acid that occurs naturally in various animal and plant sources, especially in olive oil [63]. Oleic acid is an essential fatty acid for the cardiovascular system and has anti-inflammatory, anti-cancer, and antimicrobial properties [64–67]. Similar to palmitic acid, oleic acid exhibits high oxidative stability, making its sources promising for biodiesel production [68]. The acid was found in high level (22.71%) in the TFA of the strain SUMCC 21015, while it was recoded at low levels in the other two strains (between 1.36 and 1.94%). Abdel-Wahab et al. [25] reported for the first time the presence of oleic acid and linoleic acid in the lipid profiles of three strains of *Aurantiochytrium* isolated from Red Sea mangroves in Saudi Arabia.

*Aurantiochytrium* SUMCC 21016 achieved compatible results in terms of lipid content and DHA proportion in the lipid profile, however, further biomass optimization is required. The strain produced 48.6% lipid of the dry biomass which was close to the production rate of *Aurantiochytrium* strains KRS 101 and AL-03 [25, 69]. The strain also resulted in 21.2% DHA content in the lipid profile or 10.3% in DCW. The results of DHA production were compatible with those of *Aurantiochytrium* strains KRS101, AL-03, and SY-78 and *Aurantiochytium mangrovei* B072 [25, 69–72]. Table 2 represents a comparison of our findings with those of previous studies.

**Table 2:** Dry cell weight, total fatty acids percentage, DHA%, and the media used for the different *Aurantiochytrium* strains

Species	DCW (g/L)	TFA (%DCW)	DHA (%TFA)	DHA (%DCW)	Media	References
*Aurantiochytrium* sp. KRS101	5.5	51.8	27.37	14.18	5 g/L glucose, orange peel extract, 1.5 g/L NaNO3 - Total carbohydrates 17 g/L.	[72]
*Aurantiochytrium* sp. KRS101	5	45	20	9	20 g/L glucose, 2 g/L yeast extract, 2 g/L artificial sea salt, 9 g/L KH_2_SO_4_, 10 mg/L tetracycline.	[69]
*A*. *mangrovei* Sk-02	16.9	51.18	41.6	21.29	60 g/L glucose, 10 g/L yeast extract, sea salt.	[73]
*A*. *mangrovei* B072	11.83	51.9	26.87	13.95	30 g/L glucose, 5 g yeast extract, 0.3 K_2_HPO_4_, 15 artificial sea salt, vitamins, trace elements.	[71]
*Aurantiochytrium* sp. Al-03	41	46.1	16.5	7.61	30 g/L glucose, 10 g/L peptone, 5 g/L yeast extract, 2 g/L monosodium glutamate, 0.5 g/L MgSO_4_.7H_2_O, 0.5 g/L KH_2_PO_4_, 500 mg/L chloramphenicol, in 50% seawater	[25]
*Aurantiochytrium* sp. YB-05	38.4	59.3	4.89	2.9	80 g/L glucose, 5 g/L yeast extract, 5 g/L peptone and 500 mg/L chloramphenicol.	[2]
*Aurantiochytrium* sp. SY-78	~ 16	~ 62.5	17.81	11.13	80 g/L glucose, 5 g/L yeast extract, 5 g/L peptone, 500 mg/L chloramphenicol, in 50% seawater	[70]
*A*. *limacinum* SR21	51.95	68.14	3.7 g L^− 1^ d^− 1^	---	100 g/L glucose, 3.9 g/L ammonium acetate, 4 g/L KH_2_PO_4_, 1 g/L yeast extract, 17.5 g/L artificial sea salt.	[74]
*Aurantiochytrium* sp. (SUMCC 21016)	2.04	48.6	21.21	10.31	15 g/L glucose, 5 g/L yeast extract, 5 g/L peptone, 20 g/L artificial sea salt	This study

## Conclusion

This study represents the second investigation of thraustochytrids along the Red Sea coast of Egypt and the first to study the abilities of isolated thraustochytrid strains to produce omega-3 fatty acids. Forty-one isolates of the genus *Aurantiochytrium* were successfully recovered from the Red Sea mangrove in Egypt, highlighting these habitats as valuable reservoirs of marine oleaginous protists. Based on biomass productivity and lipid accumulation, three strains (SUMCC 21014, SUMCC 21015, and SUMCC 21016) were selected for detailed morphological, physiological, and molecular phylogenetic analyses. The combined characterization confirmed their taxonomic placement and revealed notable differences in growth performance, total lipid production, and omega-3 production. Among the tested strains, SUMCC 21,016 exhibited the highest biomass, and lipid yields and showed a promising proportion of omega-3 fatty acids, representing 0.217 g/L. These findings demonstrate the potential of Red Sea mangrove-derived *Aurantiochytrium* strains as sustainable alternative sources of omega-3 fatty acids. A bottle-neck of omega-3 fatty acids production from thraustchytrids at commercial level is their low percentage compared to their biomass and their high expenses. More research needs to be done in order to lower the expenses of its production and isolating new strains from unexplored habitats that are able to produce omega-3 fatty acids at higher levels than those reported in the published articles. Overall, this work contributes to expanding knowledge of thraustochytrid diversity in the Egyptian marine ecosystems and provides a foundation for future optimization, large-scale cultivation, and potential industrial application of high-performing strains.

## Data Availability

No datasets were generated or analysed during the current study.

## Abbreviations

BI: Bayesian inference
C: N carbon-to-nitrogen ratio
CAGR: Compound annual growth rate
CI: Consistency index
DHA: Docosahexaenoic acid
DPA: Docosapentaenoic acid
DCW: Dry cell weight
EPA: Eicosapentaenoic acid
FAME: Fatty acid methyl esters
FID: Flame ionization detector
GC-FID: Gas chromatography with a flame ionization detector
GLC: Gas-liquid chromatography
GRAS: Generally recognized as safe
GYPA: Glucose-peptone-yeast extract agar medium
GYP: Broth glucose-peptone-yeast extract broth
GPY: Glucose-peptone-yeast extract medium
ML: Maximum likelihood
MP: Maximum parsimony
MUFA: Monounsaturated fatty acids
PCR: Polymerase chain reaction
PUFAs: Polyunsaturated fatty acids
RC: Rescaled consistency index
RI: Retention index
rDNA: Ribosomal DNA
SFA: Saturated fatty acids
SUMCC: Sohag university microbial culture collection
TFA: Total fatty acids
USD: United States Dollar
